# Clathrin to the rescue: Clathrin-mediated trafficking fine-tunes Arabidopsis copper tolerance

**DOI:** 10.1093/plphys/kiaf236

**Published:** 2025-06-06

**Authors:** Alicja B Kunkowska

**Affiliations:** Assistant Features Editor, Plant Physiology, American Society of Plant Biologists; PlantLab, Institute of Plant Sciences, Sant’Anna School of Advanced Studies, Pisa 56010, Italy

Copper (Cu) is an essential micronutrient for plant development, primarily absorbed from the soil (Festa and Thiele 2011). However, excessive Cu accumulation is toxic, as it leads to the overproduction of reactive oxygen species (ROS), resulting in cellular damage (Chen et al. 2022). Therefore, proper regulation of intracellular Cu levels is critical for plant survival (Xu et al. 2024). Cu homeostasis is mediated by Cu transporter proteins. Among them, heavy metal ATPase 5 (HMA5) translocates to the plasma membrane (PM) at the soil-facing side of root epidermal cells in response to elevated Cu concentrations, where it facilitates the excretion of excess Cu (Kobayashi et al. 2008; Li et al. 2017). Despite these insights, the molecular mechanisms underlying the regulation of HMA5 relocalization have remained elusive. In this issue of *Plant Physiology*, Liufan Wang and colleagues (Wang et al. 2025) identify a novel role of clathrin-mediated trafficking (CMT) in Cu detoxification by regulating HMA5 transport within Arabidopsis roots.

CMT is a key cellular mechanism in eukaryotic cells for the intracellular transport of materials. It involves the assembly of clathrin into triskelion-shaped structures, each consisting of 3 clathrin heavy chains (CHCs) and 3 light chains (CLCs), which polymerize to coat budding vesicles (Kaksonen and Roux 2018). The process is initiated by adaptor protein complexes (APs), such as AP2, which recognize cargo molecules and link them to clathrin. These adaptor complexes also help recruit clathrin to the membrane, ensuring that selected cargo is enclosed within the forming vesicle (Yamaoka et al. 2013). The involvement of CMT in responses to environmental changes has already been reported. For example, CMT regulates the uptake of iron and boron from soil via recycling and degradation of their transporter proteins (Barberon et al. 2014; Wang et al. 2017; Yoshinari et al. 2019). In these processes, clathrin recruitment to the membrane is critical. Wang and colleagues discovered that exposure to elevated Cu levels within minutes triggers a recruitment of clathrin and its adaptor proteins to both the PM and the trans-Golgi network/early endosome (TGN/EE), suggesting an immediate cellular response to Cu toxicity. This process was not observed in plants exposed to other divalent metals such as magnesium, zinc, or iron.

By combining confocal live-cell imaging, immunofluorescence, and biochemical approaches, the authors demonstrated that the increased clathrin abundance at the membranes results from rapid recruitment rather than enhanced mRNA expression or protein synthesis. Furthermore, the authors reported that mutants impaired in clathrin function or in the adaptor protein complex AP-2 (proteins that recognize cargo and recruit clathrin to the PM) showed reduced tolerance to Cu excess.

Wang and collegues (Wang et al. 2025) then established a direct link between CMT and HMA5. Genetic analysis revealed that the Cu hypersensitivity of CMT mutants mirrors that of the *hma5* knockout. Moreover, in a quadruple mutant of genes encoding the transporter along with an adaptor protein and clathrin chains (hma5 ap2µ clc2 clc3), the loss of HMA5 masks the effects of CMT deficiency, positioning HMA5 downstream of CMT in the Cu response pathway. Metal content measurements confirmed that CMT-impaired roots accumulate more Cu, further supporting the model in which CMT enhances plant tolerance via the HMA5-mediated Cu efflux pathway rather than affecting the Cu uptake pathway (Figure).

**Figure.**
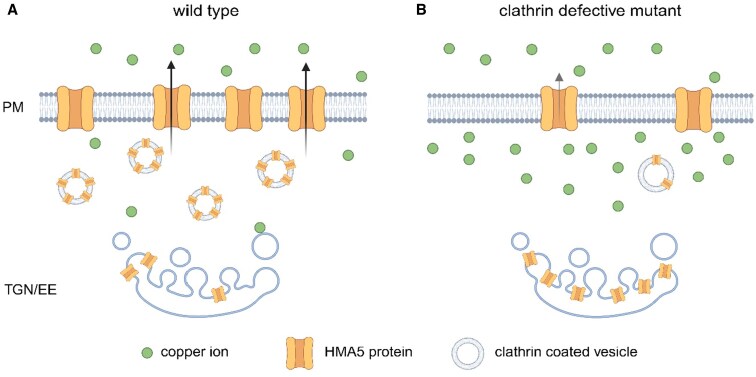
Schematic representation of the role of clathrin-mediated trafficking in regulating copper tolerance in Arabidopsis plants. **A)** In wild type plants, elevated copper levels in soil increase clathrin accumulation at the plasma membrane (PM). This promotes the transport of HMA5 via clathrin-mediated trafficking (CMT) between the *trans*-Golgi network/early endosome (TGN/EE) and the PM. HMA5 is then able to excrete excess copper, contributing to copper tolerance. **B)** In clathrin defective mutants, CMT is impaired, causing HMA5 to accumulate in the TGN/EE. As a result, excess copper cannot be efficiently excreted, leading to toxic copper buildup in root cells. Created with BioRender.com.

Live imaging of YFP-tagged HMA5 showed that CMT is required for the efficient transport of HMA5 from the TGN/EE to the PM following Cu exposure. In wild-type plants, HMA5 rapidly relocates to the soil-facing PM, where it excretes excess Cu. However, in CMT mutants, HMA5 is trapped at the TGN/EE, and its arrival at the PM is significantly delayed. Interestingly, while CMT influences the abundance of HMA5 at the PM, it does not affect its polar localization, indicating that distinct mechanisms govern polarity and trafficking efficiency. Additionally, authors identified a conserved tyrosine-based sorting motif (YXXΦ) within HMA5 that is crucial for its interaction with AP-1 and AP-2 complexes. Introducing point mutations into this motif resulted in severely impaired HMA5 transport to the PM and increased plant sensitivity to Cu.

Together, the research conducted by Wang and colleagues (Wang et al. 2025) significantly advances our understanding of how plants reorganize their endomembrane systems to cope with metal toxicity. By revealing the role of CMT in Cu detoxification, this work highlights the complexity of plant environmental adaptations. An intriguing question raised by this study is how rapidly plants respond to Cu stress. The authors note that clathrin and adaptor protein abundance at the membranes increases within just 5 min after Cu exposure, suggesting the existence of upstream Cu-sensing mechanism that triggers trafficking adaptations. Finally, Wang and colleagues' findings open new possibilities for improving plant tolerance to heavy metal stress. Engineering plants with enhanced CMT efficiency could offer strategies for breeding crops that can cope in soils contaminated by industrial activities.

## Data Availability

No data were generated or analyzed in this study.
